# Correlation between controlling nutritional status score and mortality risk in older Japanese adults diagnosed with dysphagia: a retrospective cohort study

**DOI:** 10.3389/fnut.2025.1688697

**Published:** 2026-01-12

**Authors:** Dong Wang, Peng Lv

**Affiliations:** Department of Radiology, Xi’an People’s Hospital (Xi’an Fourth Hospital), Xi’an, Shaanxi, China

**Keywords:** CONUT score, dysphagia, Japanese, malnutrition, mortality, older adults

## Abstract

**Background:**

The Controlling Nutritional Status (CONUT) score has been established as a significant prognostic indicator of clinical outcomes in diverse, critically ill, patient populations. Nevertheless, limited research has explored the relationship between CONUT score and mortality risk among older Japanese adults diagnosed with dysphagia.

**Objective:**

To evaluate the prognostic utility of the CONUT score in older Japanese adults diagnosed with dysphagia.

**Methods:**

This single-center, retrospective, observational study included data from 235 older adults diagnosed with dysphagia to quantify the prevalence of malnutrition using the CONUT score and to delineate its independent prognostic relationship with mortality. The CONUT score was categorized as normal (0–1), mild (2–4), moderate (5–8), or severe (9–12) nutritional risk. Survival analysis was performed using Kaplan–Meier curves and time-stratified Cox regression analysis. Subgroup analysis was used to examine the consistency of the results.

**Results:**

This study included 94 male and 141 female patients, with a mean (±SD) age of 82.9 ± 9.3 years, of whom 85.96% were classified as having nutritional risk according to CONUT score. Kaplan–Meier curve analysis revealed a stepwise reduction in survival with worsening malnutrition, with median survival times of 936 days (normal), 468 days (mild), 266 days (moderate), and 52 days (severe). In time-stratified Cox regression analyses, higher CONUT scores were associated with increased mortality risk [continuous CONUT score: adjusted HR 1.11 (95% CI 1.03–1.19); *p* = 0.006]. After full multivariable adjustment, a significant dose–response relationship across ordinal CONUT categories was maintained (*P* for trend = 0.008). Compared with patients with normal nutritional status, those with severe malnutrition exhibited a higher point estimate of mortality risk [adjusted HR 2.61 (95% CI 0.97–6.96); *p* = 0.055], while mild and moderate malnutrition groups showed increased but statistically non-significant risks (*p* > 0.05).

**Conclusion:**

The CONUT score shows important prognostic value for mortality risk stratification in older Japanese adults with dysphagia. A significant dose–response relationship across malnutrition categories was observed, particularly when the score was analyzed as a continuous variable, highlighting its potential utility in clinical risk assessment. These findings suggest that routine CONUT screening may help identify patients at higher nutritional risk and support timely nutritional interventions in clinical practice.

## 1 Introduction

Dysphagia is a functional impairment that predominantly affects older adults, frequently arising secondary to underlying clinical conditions that may profoundly impact quality of life and psychosocial factors (1). The estimated global prevalence of dysphagia among community-dwelling elderly individuals is approximately 20%. Complications of dysphagia, including malnutrition, fluid imbalance, and aspiration pneumonia impact 7–13% of individuals ≥ 65 years of age (2), consequently elevating the risks for extended hospitalization and mortality. A hospital-based study demonstrated that patients with dysphagia had a 1.7-fold higher mortality risk than unaffected individuals (3). The prevalence of suspected dysphagia in the general inpatient population has been reported to be 30.7% (4). Approximately 78% of stroke survivors experience dysphagia (5). Dysphagia frequently manifests in patients with disease. Globally, the prevalence of dysphagia among patients with Parkinson’s disease ranges from 40 to 80%, varying on the diagnostic approach (6). Therefore, it is crucial to prioritize the evaluation and active treatment of dysphagia.

Originally developed by de Ignacio et al. (7), the Controlling Nutritional Status (CONUT) score serves as an automated screening instrument for daily nutritional evaluation of hospitalized patients undergoing routine testing. It is calculated based on serum albumin concentration, total cholesterol concentration, total peripheral lymphocyte count, biomarkers reflecting protein metabolism, energy reserves, and immunological impairment. Increased CONUT scores are typically correlated with compromised nutritional health and diminished immune function. Recent evidence suggests that the CONUT score offers robust prognostic utility across multiple disease states. Furthermore, it has demonstrated its potential as a biomarker for predicting outcomes in diverse malignancies (8). Moreover, it demonstrates significant associations with adverse outcomes in patients with stroke, artery disease, and acute heart failure (9–11).

However, evidence-based management guidelines for dysphagia in older adults are unavailable. It is, therefore, of paramount importance to assess and predict adverse outcomes associated with dysphagia in older adults using clinical indicators. Although previous research has established the prognostic utility of the CONUT score across multiple conditions, the available evidence regarding the relationship between CONUT score and mortality risk in patients with dysphagia is insufficient and the predictive factors for mortality in this population remain to be explored. Japan presents a highly suitable cohort for this investigation, exhibiting the world’s most rapid population ageing alongside high rates of dysphagia and malnutrition among older adults (12, 13). This context provides an optimal setting to examine the CONUT score’s prognostic value. Furthermore, this study employed a well-characterised dataset from Japanese institutions, offering both statistical power and clinical applicability to justify this population focus. As such, the present study aimed to investigate whether the CONUT score was independently associated with mortality in a sample of older Japanese adults diagnosed with dysphagia.

## 2 Materials and methods

### 2.1 Data source

This study sourced data from the Dryad Digital Repository (14), an open-access platform that enables unrestricted downloading and reuse of source materials. All authors relinquished copyright to the original research data and legally authorized this secondary analysis. The dataset is readily available for download at no charge from the official Dryad database website, <https://doi.org/10.5061/dryad.gg407h1>.

### 2.2 Population study and data collection

**Figure 1:**
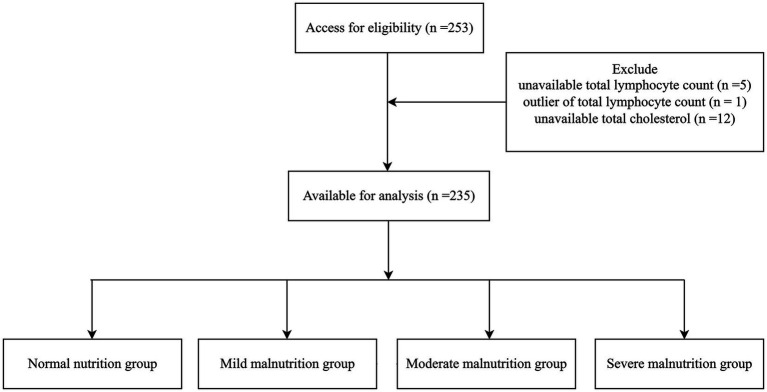
Flow diagram of patient selection. Of 253 patients assessed, 18 were excluded due to missing or abnormal data, leaving 235 for analysis. Patients were categorized into normal nutrition, mild malnutrition, moderate malnutrition, and severe malnutrition groups according to the CONUT score.

This retrospective cohort study focused on older patients diagnosed with dysphagia who underwent percutaneous endoscopic gastrostomy (PEG) or received total parenteral nutrition (TPN) between January 2014 and January 2017. The initial study enrolled 253 patients after excluding individuals with advanced-stage cancer, those requiring PEG for gastric decompression, and those who underwent PEG before January 2014. Eighteen patients with missing values or outliers were excluded. The subject exclusion parameters followed the framework outlined in Figure 1. In total, data from 235 participants were included in the study. Each enrolled patient underwent clinical dysphagia assessment through collaborative evaluation by a physician, nursing specialist, and speech-language pathologist, complemented by videofluoroscopic examination.

Secondary analysis incorporated the following clinically relevant variables: age; sex; comorbidities [cerebrovascular disorders, advanced dementia, neuromuscular conditions, aspiration pneumonia, ischemic heart disease (IHD), chronic heart failure, persistent pulmonary disease, hepatic impairment, and chronic kidney disease]; use of port/non-tunneled central venous catheter (NT-CVC)/peripherally inserted central catheter/PEG devices; oral intake recovery; laboratory parameters; body mass index; daily caloric provision; home discharge status; severe pneumonia; and sepsis. Laboratory assessments were performed within 7 days of PEG or initiation of TPN (14). Survival time after intervention initiation served as the study endpoint.

Given that older adults with dysphagia demonstrate a higher prevalence of malnutrition than their unaffected peers (15), the CONUT score was used to define nutritional risk. The CONUT score was calculated based on three routinely measured laboratory parameters available in the dataset: serum albumin concentration, total peripheral lymphocyte count, and total cholesterol level, according to the standard CONUT scoring system. CONUT scores were stratified as follows: normal (0–1); mild (2–4); moderate (5–8); or severe (9–12) (16).

### 2.3 Statistical analysis

Histograms and the Kolmogorov–Smirnov test were used to assess the normality of the variable distributions. Continuous variables that exhibited a normal distribution are expressed as mean ± standard deviation (SD), while categorical variables are expressed as frequency and percentage (%). Baseline characteristics were analyzed using one-way analysis of variance (i.e., “ANOVA”) for quantitative measures and chi-squared tests to determine intergroup differences across the nutritional status groups.

Survival distributions were visualized using Kaplan–Meier curves with log-rank testing for unadjusted group comparisons. The median follow-up time was estimated using the reverse Kaplan–Meier method based on individual survival times. To evaluate the association between predictors and mortality risk, time-stratified time-dependent Cox regression models were used as the primary analytical approach to calculate hazard ratios (HR) with corresponding 95% confidence intervals (CIs). This method was selected after formal testing of the proportional hazards (PH) assumption via Schoenfeld residuals, which revealed a significant violation of the PH assumption (global *p* = 0.002), indicating that hazard ratios were not constant over time. The follow-up period was stratified at 1200 days, a threshold identified based on visual inspection of Kaplan–Meier curves where the survival curves of the mild malnutrition group and moderate malnutrition group crossed, reflecting a critical time point for changes in hazard rate patterns. The normal nutrition group served as the reference category, with median values allocated per stratum to evaluate linear trends. CONUT score was analyzed both as a continuous and a categorical variable. The continuous model assessed the dose–response relationship, while the categorical model provided clinically relevant cut-off values and served as a sensitivity analysis to confirm robustness. Additionally, the linearity of the log-hazard relationship for continuous predictors was confirmed. To evaluate the association between the CONUT score and mortality, three analytical models with progressive adjustment were employed. Model I was a non-adjusted, univariate model used to estimate the crude hazard ratios of the CONUT score without covariate correction. Confounders for Models II and III were selected based on prior literature and their clinical relevance to mortality among older adults with dysphagia. Model II was adjusted for age, sex, cerebrovascular disease, severe dementia, aspiration pneumonia, and ischemic heart disease (17–20). Model III further included non-tunneled central venous catheter use, percutaneous endoscopic gastrostomy (PEG), oral intake recovery status, and hemoglobin level, which were identified as key clinical variables in the original dataset (14) and supported by previous studies as indicators of nutritional intervention (21), swallowing function, and overall health condition.

Interactions across the subgroups were assessed using the likelihood ratio test. Subgroup and interaction analyses used the same clinically relevant variables as those included in the multivariable time-stratified Cox models (age, sex, cerebrovascular disease, severe dementia, aspiration pneumonia, ischemic heart disease, PEG, and non-tunneled central venous catheter use). These subgroups were selected to examine whether the association between nutritional status and mortality was consistent across biologically and clinically meaningful categories. As this study was a secondary exploratory analysis based on publicly available data, no prespecified effect-modification hypotheses were defined. Subgroup analyses were therefore intended to evaluate the robustness and consistency of the main findings rather than for confirmatory hypothesis testing. Given the modest sample size and number of events, these interaction tests were underpowered and should be interpreted as exploratory. All statistical tests were two-sided and differences with *p* < 0.05 were considered to be significant.

Statistical computations were performed using R version 4.2.2 (R Core Team; R Foundation for Statistical Computing, Vienna, Austria <http://www.R-project.org>) alongside Free Statistics platform version 2.1.1 (developed by Beijing FreeClinical Medical Technology Co., Ltd., China).

## 3 Results

### 3.1 Baseline patient characteristics

Data from 235 patients (40% male) with a mean (± SD) age of 82.9 ± 9.3 years, were analyzed. The CONUT score-based stratification identified 33 (14.0%) patients as normal, 77 (32.8%) as mild, 82 (34.9%) as moderate, and 43 (18.3%) as severe. Baseline characteristics of the groups stratified according to CONUT score are summarized in Table 1. There were significant differences among the 4 groups in terms of age, cerebrovascular disease, severe dementia, aspiration pneumonia, hemoglobin, PEG, NT-CVC, and marital status (all *p* < 0.05).

**Table 1:** Baseline characteristics of patients.

Variables	Total (*n* = 235)	1 (*n* = 33)	2 (*n* = 77)	3 (*n* = 82)	4 (*n* = 43)	*p*
Age, Mean ± SD	82.9 ± 9.3	75.4 ± 13.4	82.1 ± 8.8	85.1 ± 7.3	85.7 ± 6.6	< 0.001
Sex, *n* (%)						0.107
Male	94 (40.0)	8 (24.2)	28 (36.4)	39 (47.6)	19 (44.2)	
Female	141 (60.0)	25 (75.8)	49 (63.6)	43 (52.4)	24 (55.8)	
CI, *n* (%)	125 (53.2)	27 (81.8)	46 (59.7)	35 (42.7)	17 (39.5)	< 0.001
Dement, *n* (%)	97 (41.3)	5 (15.2)	26 (33.8)	43 (52.4)	23 (53.5)	< 0.001
Asp., *n* (%)	89 (37.9)	5 (15.2)	28 (36.4)	38 (46.3)	18 (41.9)	0.018
IHD, *n* (%)	42 (17.9)	4 (12.1)	11 (14.3)	15 (18.3)	12 (27.9)	0.223
Hemoglobin, Mean ± SD	11.0 ± 2.0	12.8 ± 1.5	11.3 ± 1.8	10.9 ± 1.8	9.1 ± 1.7	< 0.001
Oral, *n* (%)	14 (6.0)	4 (12.1)	4 (5.2)	4 (4.9)	2 (4.7)	0.518
PEG, *n* (%)	168 (71.5)	32 (97)	58 (75.3)	57 (69.5)	21 (48.8)	< 0.001
NT. CVC, *n* (%)	23 (9.8)	0 (0)	6 (7.8)	7 (8.5)	10 (23.3)	0.008
Status, *n* (%)						< 0.001
Death	130 (55.3)	6 (18.2)	40 (51.9)	50 (61)	34 (79.1)	
Alive	105 (44.7)	27 (81.8)	37 (48.1)	32 (39)	9 (20.9)	
CONUT, Mean ± SD	5.1 ± 3.1	0.5 ± 0.5	3.2 ± 0.8	6.3 ± 1.1	9.9 ± 1.0	< 0.001

CI, cerebrovascular diseases; Dement, severe dementia; Asp., aspiration pneumonia; IHD, ischemic heart disease; PEG, percutaneous endoscopic gastrostomy; TPN, total parenteral nutrition; Oral, oral intake recovery; PNI, prognostic nutritional index.

### 3.2 Kaplan–Meier curve analysis

**Figure 2:**
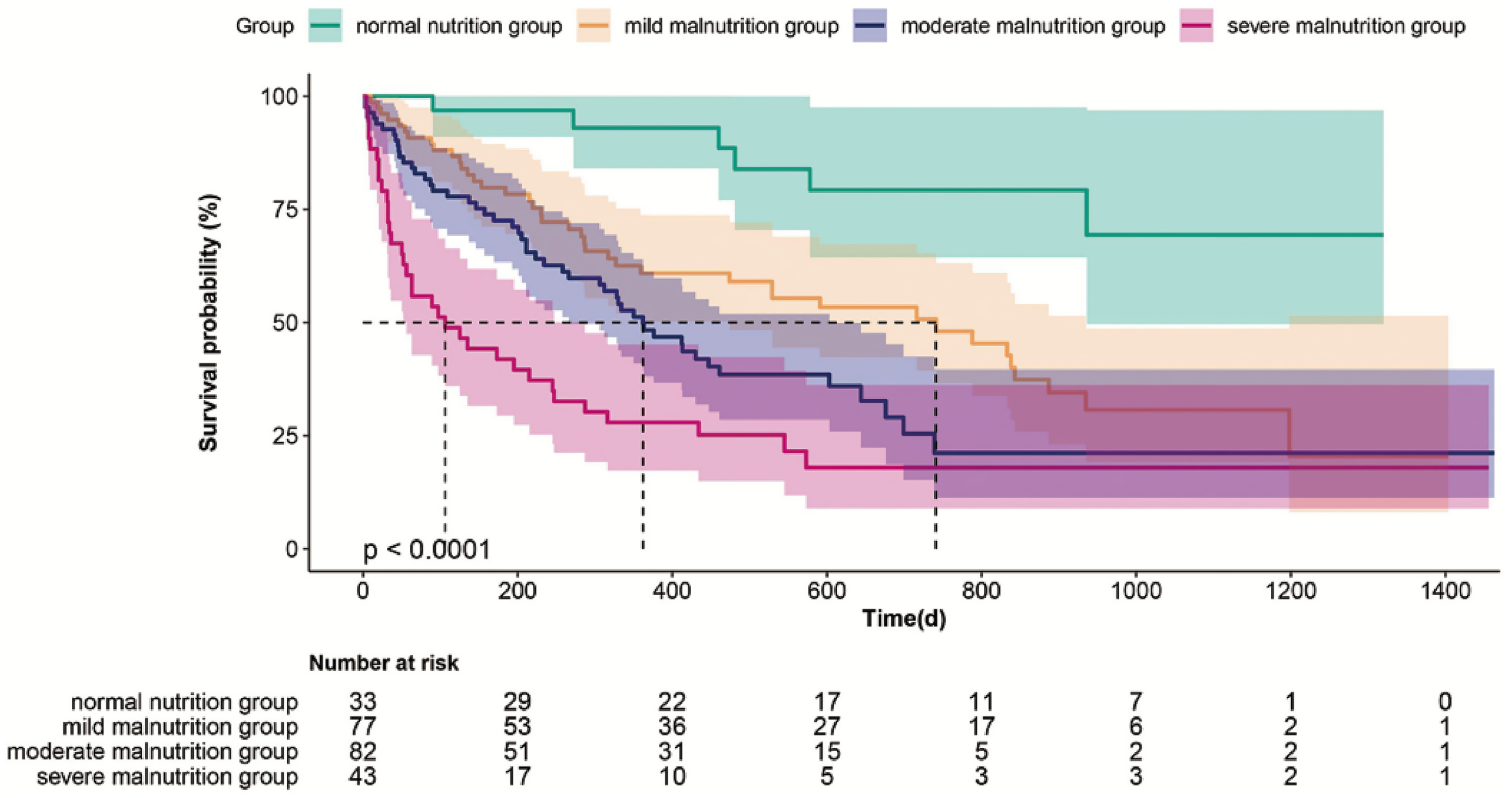
Kaplan–Meier survival curves stratified by nutritional status assessed using the CONUT score. Survival probability declined progressively with worsening nutritional status (log-rank test, *p* < 0.0001). Numbers at risk are presented below the curves.

The maximum observed follow-up time was 1,463 days (approximately 48 months). The median follow-up duration was 312 days (interquartile range: 112–636 days). Kaplan–Meier curve analysis demonstrated a progressive decline in survival probability corresponding to increasing severity of malnutrition, with the severe malnutrition group exhibiting a significantly higher cumulative mortality risk than the mild, moderate, and normal nutrition groups (log-rank test, *p* < 0.0001) (Figure 2). The median survival times in the normal, mild, moderate, and severe malnutrition groups were 936, 468, 266, and 52 days, respectively.

### 3.3 Association between CONUT score and mortality in various models

HRs and corresponding 95% CIs for mortality risk in patients with dysphagia according to CONUT score are reported in Table 2. Due to the violation of the proportional hazards assumption (Schoenfeld residuals global *p* = 0.002) and the observed crossing of survival curves between the mild and moderate malnutrition groups at 1200 days, all analyses were performed using time-stratified time-dependent Cox regression with the follow-up period stratified at 1200 days to account for this time-dependent change in hazard patterns. Four analytical approaches were used: Model I incorporated CONUT score as continuous/categorical variables without covariate correction; Model II included demographic (age, sex) and comorbidity adjustments (cerebrovascular disorders, advanced dementia, aspiration pneumonia, IHD); Model III integrated comprehensive covariates including age, sex, IHD, cerebrovascular disorders, advanced dementia, aspiration pneumonia, PEG, hemoglobin, NT-CVC, and oral intake recovery. These analytical outcomes are detailed in Table 2. Univariate time-stratified Cox analysis revealed that CONUT score, as a continuous variable, was significantly associated with mortality risk (adjusted HR 1.11 [95% CI 1.03–1.19]; *p* = 0.006). After adjusting for covariates in the multivariable time-stratified Cox model (stratified at 1200 days to address the crossing survival curves), a significant dose–response relationship was observed across ordinal CONUT categories (*P* for trend = 0.008). More specifically, compared with the normal nutrition group, severe malnutrition was associated with a higher point estimate of mortality risk [adjusted HR 2.61 (95% CI 0.97–6.96); *p* = 0.055], although this association did not reach statistical significance. Similarly, mild and moderate malnutrition did not reach statistical significance [mild, HR 1.88 (95% CI 0.77–4.59), *p* = 0.163; moderate, HR 2.10 (95% CI 0.85–5.20), *p* = 0.108]; however, their point estimates aligned with the graded risk increase indicated by the trend test. The wide confidence intervals may reflect limited power and potential type II error in these subgroups.

**Table 2:** Univariate and multivariate time-stratified Cox regression models for mortality.

Variable	Model I		Model II		Model III	
	HR (95%CI)	*p*	HR (95%CI)	*p*	HR (95%CI)	*p*
CONUT, continuous						
Per 1-score increment	1.23 (1.16 ~ 1.3)	<0.001	1.18 (1.1 ~ 1.25)	<0.001	1.11 (1.03 ~ 1.19)	0.006
CONUT, categorical						
Normal	1 (Ref)		1 (Ref)		1 (Ref)	
Mild	3.67 (1.56 ~ 8.67)	0.003	2.73 (1.15 ~ 6.51)	0.023	1.88 (0.77 ~ 4.59)	0.163
Moderate	5.53 (2.36 ~ 12.97)	<0.001	2.96 (1.23 ~ 7.13)	0.016	2.10 (0.85 ~ 5.20)	0.108
Severe	9.51 (3.98 ~ 22.73)	<0.001	5.58 (2.29 ~ 13.57)	<0.001	2.61 (0.97 ~ 6.96)	0.055
P for trend		<0.001		<0.001		0.008

Model I: non-adjusted.

Model II: adjusted for age, sex, cerebrovascular diseases, severe dementia, aspiration pneumonia, ischemic heart disease.

Model III: adjusted for age, sex, cerebrovascular diseases, severe dementia, aspiration pneumonia, ischemic heart disease, non-tunneled central venous catheters, percutaneous endoscopic gastrostomy, oral intake recovery, hemoglobin.

CONUT, Controlled Nutritional Status Score.

### 3.4 Subgroup analyses

**Figure 3:**
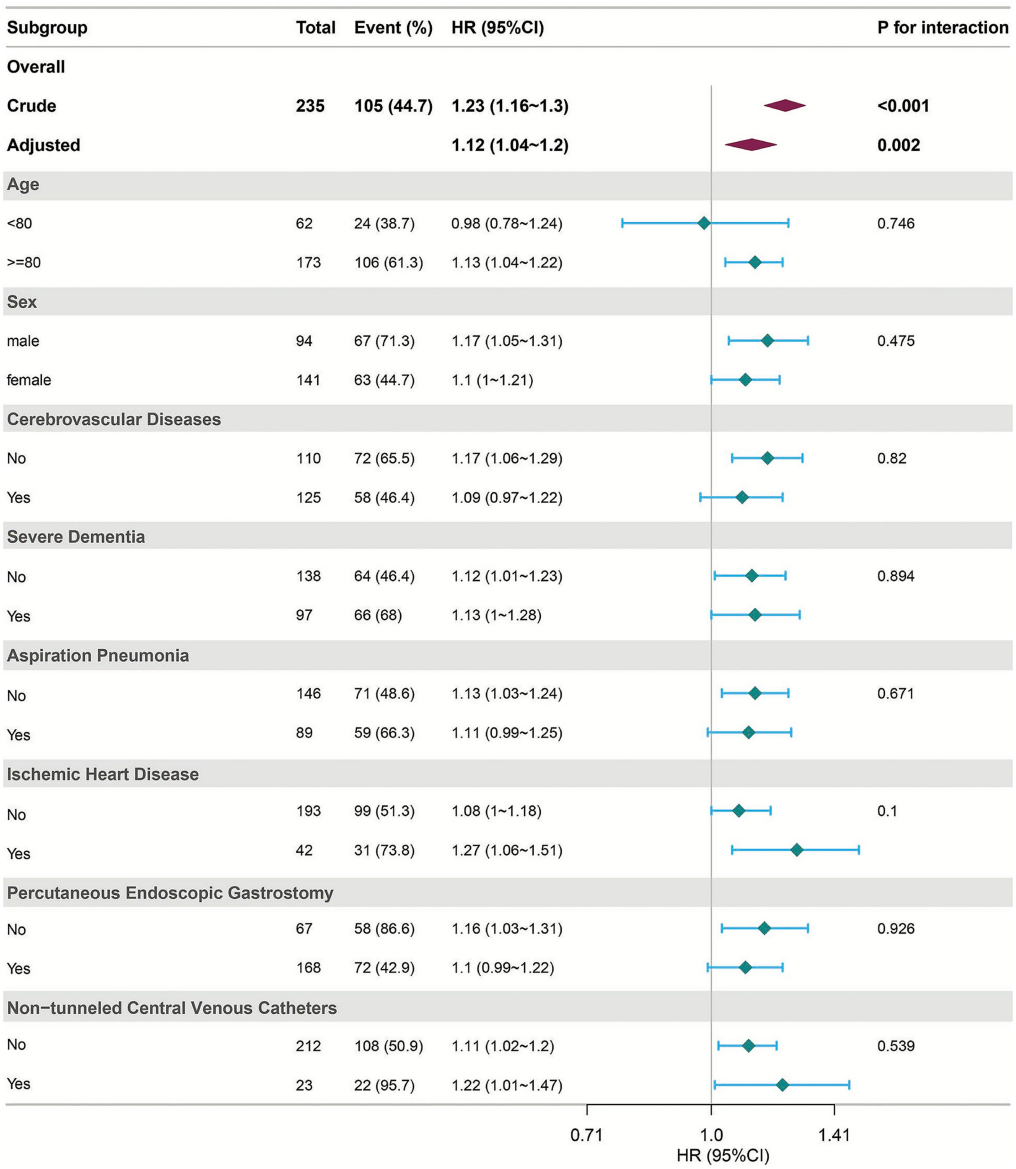
Forest plot showing hazard ratios (HRs) with 95% confidence intervals (CIs) for overall and subgroup analyses. Both crude and adjusted models are presented. Subgroup analyses were conducted according to age, sex, cerebrovascular diseases, severe dementia, aspiration pneumonia, ischemic heart disease, percutaneous endoscopic gastrostomy, and non-tunneled central venous catheters. *p* values for interaction are shown on the right.

Subgroup and interaction analyses were used to evaluate the robustness of CONUT score mortality associations in dysphagia (Figure 3). Following full adjustment for covariates (age, sex, IHD, cerebrovascular disorders, advanced dementia, aspiration pneumonia, PEG, hemoglobin, NT-CVC, and oral intake recovery), categorical subgrouping variables were systematically omitted from the covariate sets during stratified analyses to prevent duplication. The results indicated that the risk estimates for incident mortality were consistently comparable across the various subgroups, with all interaction *p*-values exceeding 0.05.

## 4 Discussion

To our knowledge, this is the first retrospective cohort study to demonstrate a consistent dose–response association between CONUT-assessed malnutrition severity and mortality in older Japanese adults diagnosed with dysphagia. The significant trend test (*p* = 0.008) and progressively reduced survival times (median survival 936 days for normal nutrition → 52 days for severe malnutrition) support a graded association between worsening malnutrition and mortality risk. Consistently, each one-point increase in CONUT score was associated with an 11.1% higher risk of mortality [adjusted HR 1.11 (95% CI 1.03 ~ 1.19); *p* = 0.006]. Importantly, while severe malnutrition (CONUT score ≥ 9) exhibited a higher point estimate of mortality risk [adjusted HR 2.61 (95% CI 0.97 ~ 6.96); *p* = 0.055] in the fully adjusted model, its 2.6-fold risk elevation aligned with the graded risk increase indicated by the trend test. These findings highlight the necessity of regular nutritional screening in older patients with dysphagia to improve outcomes (22).

Both identifiable disease states and physiological aging contribute to the increased prevalence of dysphagia in population-based studies (23). Dysphagia can cause clinically relevant complications that substantially affect patient health, nutritional equilibrium, and quality of life. Compromised swallowing safety results in the development of aspiration pneumonia in 50% of affected individuals, concurrently impairing patients’ ability to intake all essential calories and fluids necessary for sustaining optimal nutrition and hydration homeostasis (24). A study involving community-dwelling older adults in China revealed that participants with dysphagia exhibited a significantly higher risk for malnutrition than those without swallowing difficulties (25). These complications increase mortality in patients with dysphagia (23, 26, 27).

Our findings align with those of previous studies linking the CONUT score to poor outcomes in chronic diseases. Compared with the Prognostic Nutritional Index (PNI), Geriatric Nutritional Risk Index (GNRI), and Mini Nutritional Assessment–Short Form (MNA-SF), CONUT shows distinct advantages. Unlike GNRI, which depends on body weight (28), or MNA-SF, which includes subjective items (29), CONUT relies solely on objective laboratory parameters—albumin, lymphocyte count, and total cholesterol—allowing accurate evaluation even in patients with dysphagia or cognitive impairment. In idiopathic pulmonary arterial hypertension, Zhang et al. (30) reported superior predictive ability of CONUT versus GNRI and PNI, reflecting its integrated assessment of protein reserves, immune competence, and energy metabolism. Similarly, in esophageal cancer, Yoon et al. (31) demonstrated comparable or higher prognostic accuracy of CONUT. Previous studies have demonstrated that malnourished (CONUT score ≥2) patients have an increased risk for major adverse cardiovascular events and reported that the CONUT score as an independent risk factor for all-cause mortality in patients with coronary artery disease (9). Kato et al. (10) identified an elevated CONUT score as a predictor of increased inpatient mortality and infection susceptibility among patients admitted for acute heart failure. Originally functioning as a nutritional screening instrument (7), the CONUT score has gained recognition as a validated prognostic metric for diverse malignancies including gynecological, gastric, colorectal, and hepatocellular carcinomas (32–35). Contemporary research has consistently confirmed their superiority over single biomarkers for predicting oncological outcomes (36, 37).

Recent evidence has revealed that the prognostic relevance of the CONUT score extends to multiple inflammatory conditions, including rheumatoid arthritis and ulcerative colitis (38, 39), suggesting its role in capturing systemic metabolic inflammation. In our study population, the CONUT score, particularly when analyzed as a continuous variable, was an independent risk factor for mortality in older Japanese adults with dysphagia, even after adjusting for potential confounders (*p* = 0.006), with survival distributions in Figure 2 diverging significantly across the nutritional risk categories (*p* < 0.0001). This underscores its robustness in geriatric populations with complex comorbidities, in whom traditional nutritional assessments may fail due to confounding by inflammation or organ dysfunction. Mechanistically, CONUT score’s prognostic utility stems from the synergy of its components: serum albumin reflects protein reserves and systemic inflammation (40, 41), lyphocytopenia indicates impaired immune competence (42), and low cholesterol signifies depleted caloric reserves (43). This integrated assessment is particularly adept at identifying the complex malnutrition-inflammation profile often seen in frail older adults with dysphagia. Nevertheless, we acknowledge that our work mainly provides confirmatory evidence within a specific high-risk population (older adults with dysphagia), rather than introducing a novel biomarker or methodological innovation.

Previous investigations have not validated the prognostic utility of the CONUT score in geriatric dysphagia populations, a high-risk cohort frequently burdened with rapid nutritional deterioration and fatal sequelae. Our study establishes a dose-dependent relationship between CONUT-assessed malnutrition severity and mortality among older Japanese adults with dysphagia. Although categorical associations did not all reach statistical significance in the most stringent model (Model III), severe malnutrition (CONUT score ≥ 9) was associated with a 2.6-fold higher point estimate for mortality risk (*p* = 0.055) and a critically short median survival of 52 days, suggesting potential clinical relevance that warrants early nutritional attention. To translate this finding into practice, we propose a structured clinical pathway for CONUT integration. Upon admission, patients with dysphagia should be screened using the CONUT score. This score can then guide triage of nutritional interventions. For patients with severe malnutrition (CONUT ≥9), a proactive protocol should be triggered, including consultation with a multidisciplinary nutrition support team (involving a physician, dietitian, and speech-language pathologist) and consideration of early enteral nutrition (e.g., within 48 h) to bypass swallowing difficulties and ensure adequate nutrient delivery. For those with mild-to-moderate risk (CONUT score 2–8), regular proactive surveillance (e.g., weekly CONUT reassessment) should be established, with nutritional support (such as oral nutritional supplements and diet texture modification according to IDDSI frameworks) escalated if their scores deteriorate (22). This framework remains effective across diverse clinical settings. Subgroup analyses confirmed no significant interaction effects of demographic characteristics (age/sex), comorbidities (cerebrovascular disorders, advanced dementia, aspiration pneumonia, and IHD), or nutritional interventions (PEG/TPN). The consistency of the dose–response relationship (*P* for trend = 0.008) across these models further supports the prognostic utility of the CONUT score. However, subgroup and interaction analyses were underpowered due to the modest sample size and should therefore be interpreted as exploratory and hypothesis-generating. The potential of the CONUT score as a “pan-comorbidity” triage tool is noteworthy. It offers a standardized approach to nutritional risk stratification, even in patients with advanced multimorbidity, where traditional nutrition indices may fall short. Notably, the three biomarkers used to calculate the CONUT score are already routinely assessed in clinical practice, meaning their inclusion imposes no additional financial burden on healthcare systems. This simplicity makes the CONUT score a highly feasible, cost-effective tool for routine use in clinical settings.

This study was conducted at a single Japanese center, and cultural, dietary, and healthcare system-specific factors could limit the generalizability of our findings. Japan’s older adults typically have a high fish-based protein intake—relevant to nutritional status—and benefit from a structured long-term care system (44, 45). These contextual characteristics may have influenced participants’ baseline nutritional profiles and the observed association between CONUT score and mortality. Consequently, the prognostic performance of the CONUT score observed in this cohort may not be directly applicable to populations with different dietary habits or healthcare structures.

In summary, nutritional risk stratification based on the CONUT score has the potential to transform malnutrition from a “silent killer” into a modifiable risk factor. By enabling early and tailored nutritional interventions, this approach may reduce preventable mortality and shift geriatric nutritional care from a reactive to a proactive, preventive model. This shift is crucial in improving the overall quality of care for elderly patients with dysphagia, who are at heightened risk for malnutrition-related complications.

The present study, however, had several limitations that warrant careful consideration. First, this was a single-center retrospective study conducted in Japan using secondary data from an existing dataset. In line with reviewer concerns, the incremental scientific contribution is therefore modest, as the present work primarily extends existing knowledge of CONUT prognostic value to a specific clinical subgroup rather than establishing new mechanistic pathways or proposing novel analytic frameworks. Such a design may introduce selection bias, limit control over data collection (risking inaccuracies or residual confounding), and reduce external validity. Future multi-center prospective studies across diverse populations and healthcare systems are warranted to confirm and extend these findings. Second, the relatively small sample size restricted statistical power and particularly limited the ability to assess subgroup differences or interaction effects. The wide confidence intervals observed for mild and moderate malnutrition groups likely reflect limited power and potential type II error. These analyses should therefore be regarded as exploratory and hypothesis-generating. Although we endeavored to adjust for all pertinent confounders within the multivariate model, it remains possible that unaccounted residual factors (e.g., nutritional patterns and household economics) may exist, possibly inflating the identified relationships beyond the actual causal effects. Additionally, while model assumptions were formally tested and an appropriate time-stratified Cox approach was applied to accommodate time-varying hazards, this requirement itself indicates that hazard patterns may not remain stable over time and highlights the need for external validation. Third, while all PEG/TPN recipients had clinically confirmed severe dysphagia, patients with mild or moderate dysphagia who were managed conservatively were excluded from the analysis. This restriction may limit the generalizability of our findings, as the prognostic value of the CONUT score in less severe dysphagia remains uncertain and could differ from that observed in patients with advanced disease. Considering these limitations, conducting well-designed, multicenter, controlled trials that include the full spectrum of dysphagia severity is imperative to validate our findings and their broader applicability.

## 5 Conclusion

Our findings indicate that the severity of malnutrition—assessed according to CONUT score—is associated with an increased risk of mortality in older Japanese adults with dysphagia. A significant dose–response relationship across CONUT categories was observed, particularly when the score was analyzed as a continuous variable, supporting its potential value for nutritional risk stratification. Routine CONUT assessment may help identify patients at higher risk and inform timely interventions in clinical practice.

## Data Availability

The datasets presented in this study can be found in online repositories. The names of the repository/repositories and accession number(s) can be found at: Dryad Digital Repository, https://doi.org/10.5061/dryad.gg407h1.
